# Author Correction: Simulation-based reconstruction of global bird migration over the past 50,000 years

**DOI:** 10.1038/s41467-020-15528-x

**Published:** 2020-03-31

**Authors:** Marius Somveille, Martin Wikelski, Robert M. Beyer, Ana S. L. Rodrigues, Andrea Manica, Walter Jetz

**Affiliations:** 1 Ecology and Evolutionary Biology Department, Yale University, New Haven, CT USA; 2 Center for Biodiversity and Global Change, Yale University, New Haven, CT USA; 3 Department of Migration, Max Planck Institute of Animal Behavior, Radolfzell, Germany; 4 BirdLife International, The David Attenborough Building, Cambridge, UK; 5 Centre for the Advanced Study of Collective Behaviour, University of Konstanz, Konstanz, Germany; 6 Department of Zoology, University of Cambridge, Cambridge, UK; 7 Centre d’Ecologie Fonctionnelle et Evolutive CEFE UMR 5175, CNRS-Université de Montpellier-Université Paul-Valéry Montpellier—EPHE, Montpellier, France

**Keywords:** Animal migration, Biogeography, Ecological modelling, Palaeoecology

Correction to: *Nature Communications* 10.1038/s41467-020-14589-2, published online 18 February 2020.

The original version of this Article contained an error in the legend of Fig. 2 and Supplementary Figs. 2 and 8–16, each of which incorrectly stated ‘The global patterns were computed as the predicted richness in the past, i.e. predicted number of species per hexagon: 10,000 years before present (**a**, **b**); 20,000 BP (**c**, **d**); and 50,000 BP (**e**, **f**), minus the predicted richness in the present. BP before present. Red areas had more species than today, blue areas fewer’. The correct version reads ‘The global patterns were computed as the predicted richness in the present minus the predicted richness in the past, i.e. predicted number of species per hexagon: 10,000 years before present (**a**, **b**); 20,000 BP (**c**, **d**); and 50,000 BP (**e**, **f**). BP before present. Red areas had fewer species in the past than today, blue areas more’. This has been corrected in the PDF and HTML versions of the Article, and the HTML has been updated to include a corrected version of the Supplementary Information.

