# Supplementary material for: Simulation-based reconstruction of global bird migration over the past 50,000 years
Source: Nat Commun. 2020 Feb 18;11:801. doi: 10.1038/s41467-020-14589-2 (PMC7028998; doi:10.1038/s41467-020-14589-2)
Supplement: Supplementary file 1 — Supplementary Information [file 41467_2020_14589_MOESM1_ESM.pdf]

# Simulation-based reconstruction of global bird migration over the past 50,000 years

## Supplementary Information

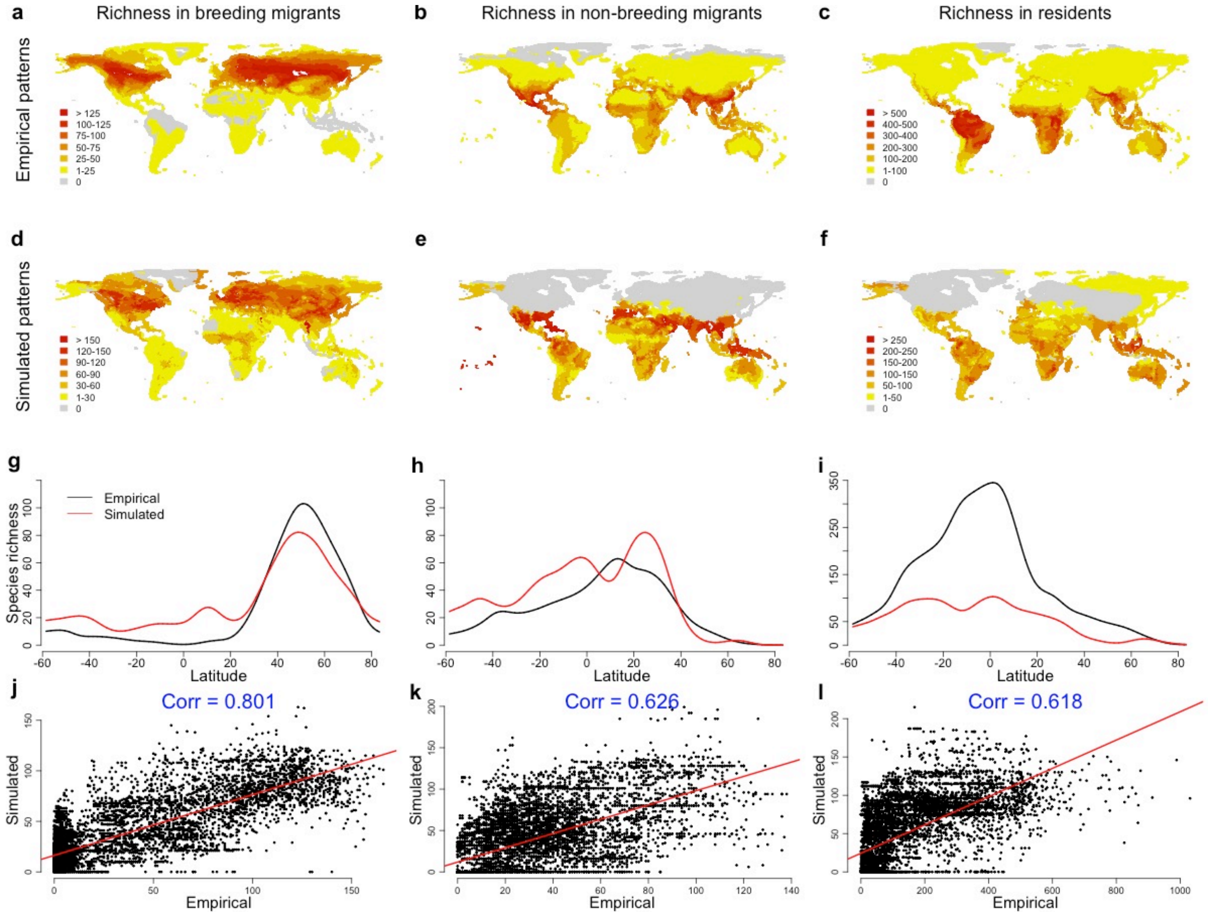


**Supplementary Figure 1:** Predicting the current global seasonal distribution of birds. This panel of figures shows the predictions from the best model for the three patterns associated with the global seasonal distribution of birds: richness in breeding migrants (**a**,**d**,**g**,**j**), richness in non-breeding migrants (**b**,**e**,**h**,**k**) and richness in residents (**c**,**f**,**i**,**l**). **a**–**c** Empirical spatial patterns. **d**–**f** Simulated spatial patterns. **g**–**i** Empirical and simulated latitudinal trends, obtained using Nadaraya-Watson kernel regression estimates (using the ksmooth function from the stats package in R). **j**–**l** Scatterplots of relationship between empirical and simulated patterns (each point corresponds to an hexagon on the spatial grid).

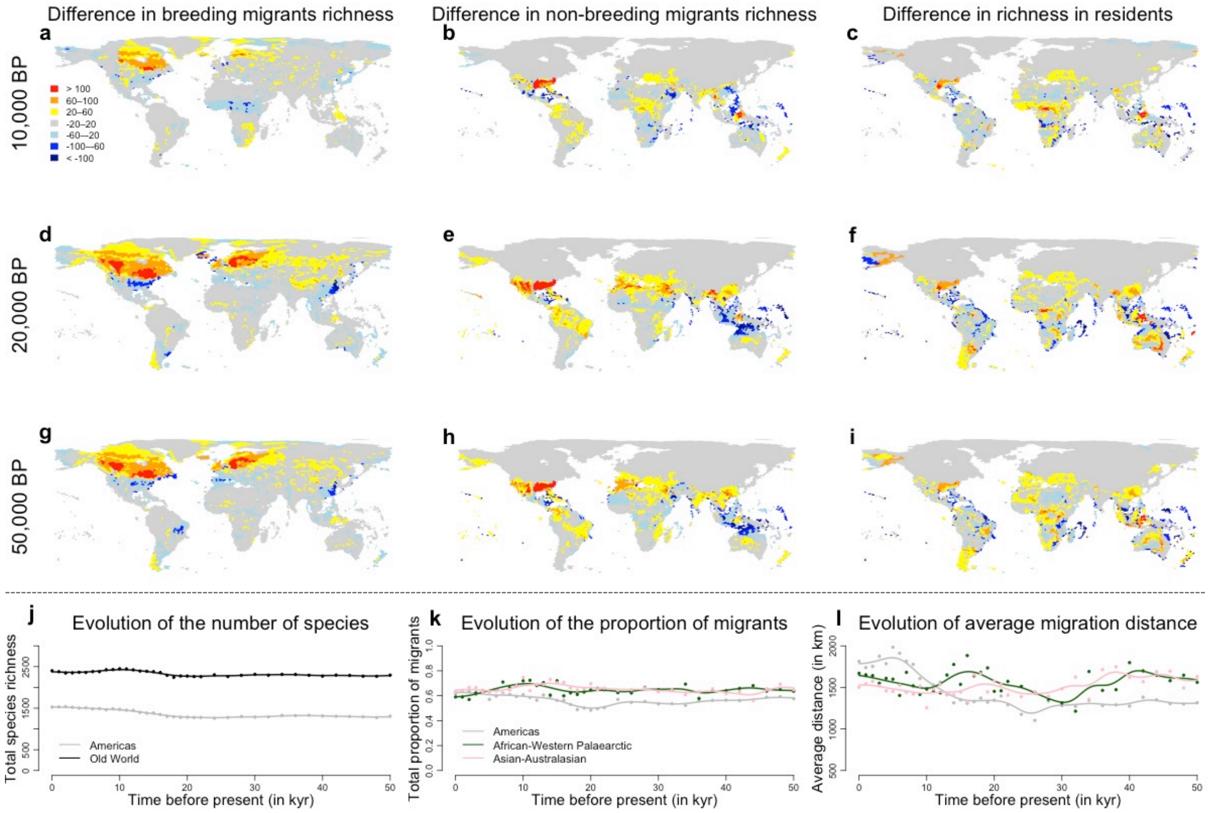


**Supplementary Figure 2:** Reconstruction of the global seasonal distribution of birds using the best-fit model. **a–i** Predicted contrast between global seasonal patterns of bird diversity (breeding migrants, non-breeding migrants and residents) over the past 50,000 years, compared to current patterns. The patterns were computed as the predicted richness in the past, i.e., predicted number of species per hexagon: 10,000 years before present (**a**–**c**); 20,000 BP (**d**–**f**); and 50,000 BP (**g**–**i**), minus the predicted richness in the present. Red areas had more species than today, blue areas fewer. **j** Predicted evolution of the total number of simulated migratory bird species, which remains largely stable over the past 50,000 years, with some slight decrease around 15,000 years before present. **k** Predicted evolution of the proportion of simulated migratory bird species. **l** Predicted evolution of the average distance between breeding and non-breeding grounds for migrant species, computed as the great circle distance between the centroids of the seasonal ranges. These simulated time series (**j**–**l**) are shown for the Americas (in grey) and the Old World (in black), the former being separated into the African–Western Palaearctic (centroids of non-breeding ranges of simulated species with a longitude < 60°E) and Asian-Australasian (centroids of non-breeding ranges of simulated species with a longitude >= 60°E) regions in panels **k**,**l**. BP: Before Present; 1kyr = 1,000 years.

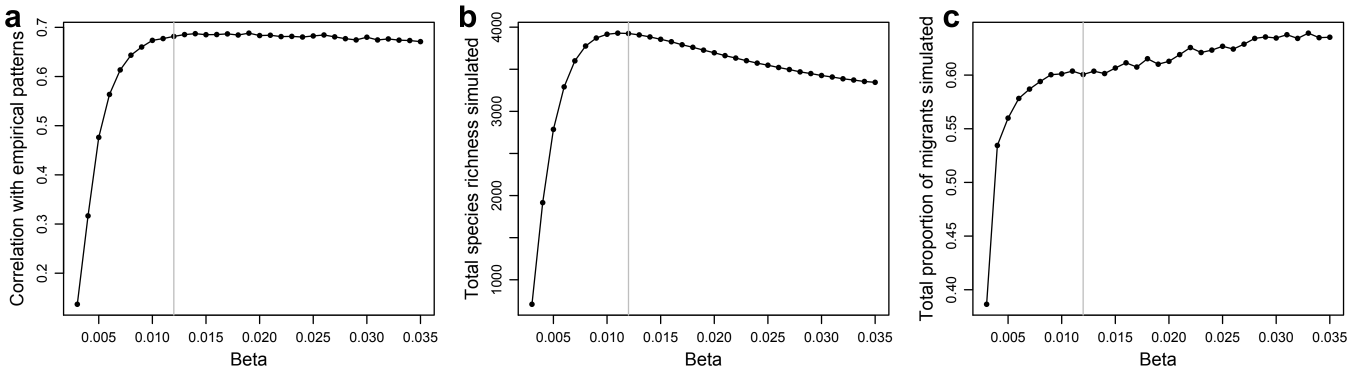


**Supplementary Figure 3:** Model calibration. Relationship between $\beta$, the only free parameter in the model, and the correlation between empirical and simulated patterns (sum of the correlations for the richness in breeding migrants, non-breeding migrants and residents) (**a**); the total number of species simulated (**b**), and the total proportion of simulated species that are migrants (**c**). The grey vertical lines indicate the value for $\beta$ that was selected for back-casting the global seasonal distribution of birds.

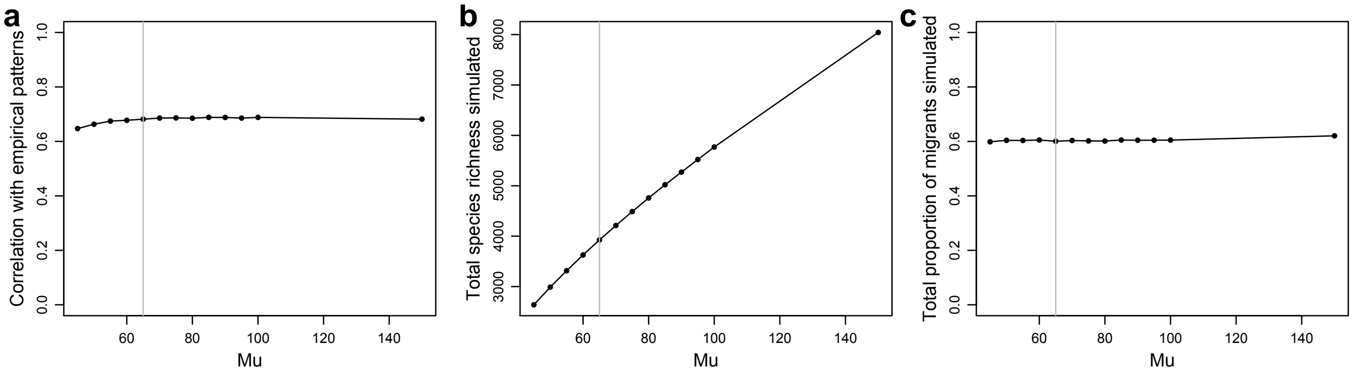


**Supplementary Figure 4:** Sensitivity of model outputs to variations in the adjustment of energy supply. Relationship between $\mu$, the parameter converting NPP values to units of energy supply, and the correlation between empirical and simulated patterns (sum of the correlations for the richness in breeding migrants, non-breeding migrants and residents) (**a**); the total number of species simulated (**b**), and the total proportion of simulated species that are migrants (**c**). The grey vertical lines indicate the fixed value for $\mu$ that was used for the main analyses.

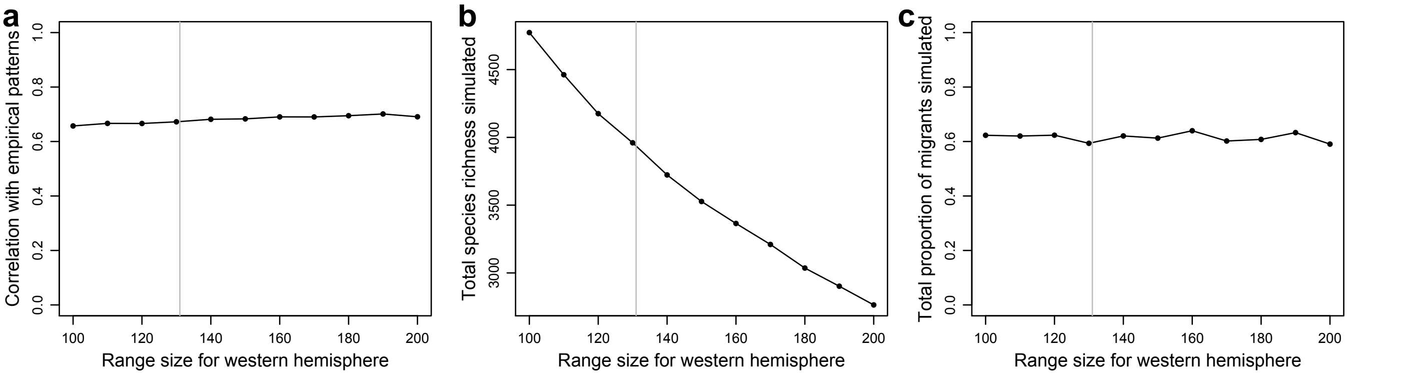


**Supplementary Figure 5:** Sensitivity of model outputs to variations in the size of the simulated range options. Relationship between the range size of simulated range options and the correlation between empirical and simulated patterns (sum of the correlations for the richness in breeding migrants, non-breeding migrants and residents) (**a**); the total number of species simulated (**b**), and the total proportion of simulated species that are migrants (**c**). The grey vertical lines indicate the range size value used for the main analyses, estimated as the median value in a global dataset of avian species range maps (BirdLife International and NatureServe, 2012). The x-axes in this figure indicate the range size for the western hemisphere, for which there was a corresponding range size values of $x$ + 50 in the eastern hemisphere.

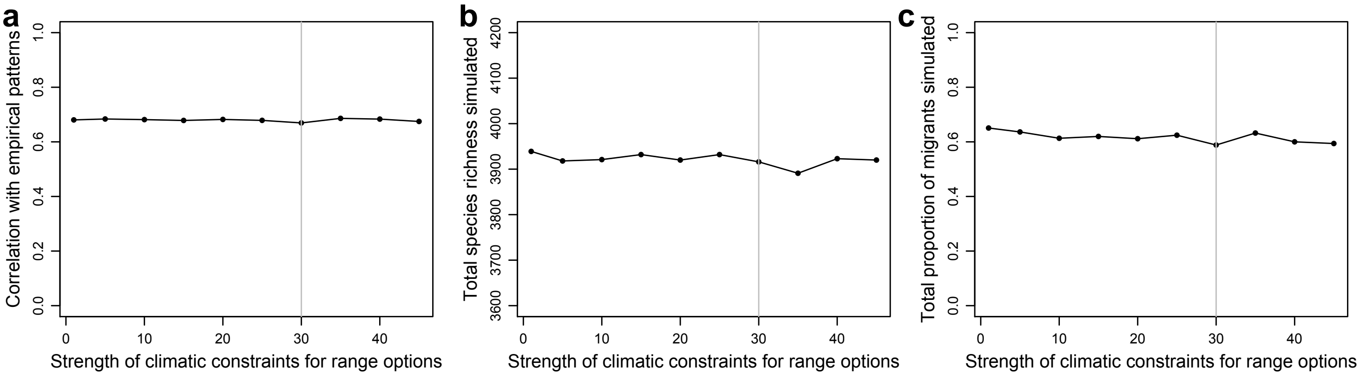


**Supplementary Figure 6:** Sensitivity of model outputs to variations in the strength of the climatic constraints when simulating range options. Relationship between the value of $x$ in $2(d + 1)^{-x}$, which defines the probability of selection for neighbouring hexagons in the spreading dye algorithm used to simulate range options (see section on virtual species range options in Methods), and the correlation between empirical and simulated patterns (sum of the correlations for the richness in breeding migrants, non-breeding migrants and residents) (**a**);

the total number of species simulated (**b**), and the total proportion of simulated species that are migrants (**c**). The grey vertical lines indicate the value used for the main analyses.

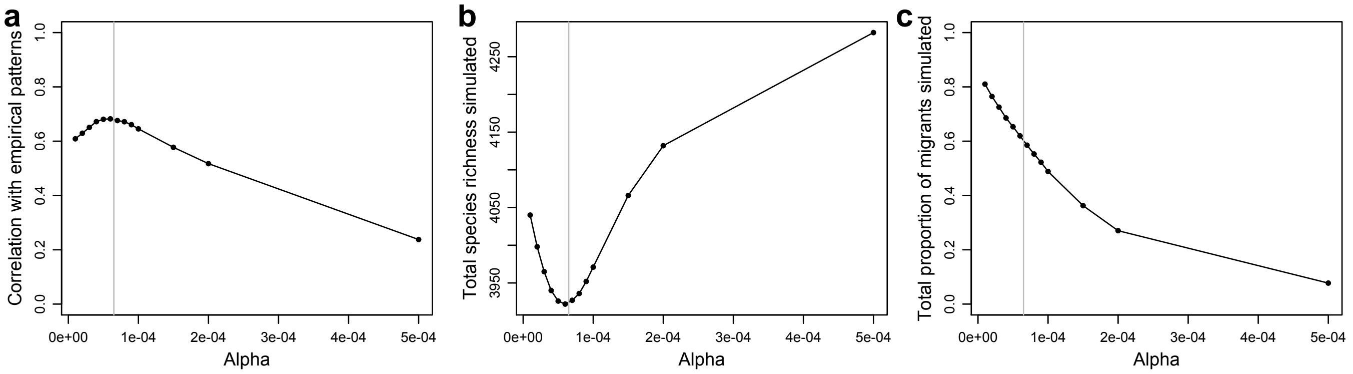


**Supplementary Figure 7:** Sensitivity of model outputs to variations in migration cost. Relationship between $\alpha$, the parameter associated with the cost of migration, and the correlation between empirical and simulated patterns (sum of the correlations for the richness in breeding migrants, non-breeding migrants and residents) (**a**); the total number of species simulated (**b**), and the total proportion of simulated species that are migrants (**c**). The grey vertical lines indicate the value for $\alpha$ that was estimated directly from the literature.

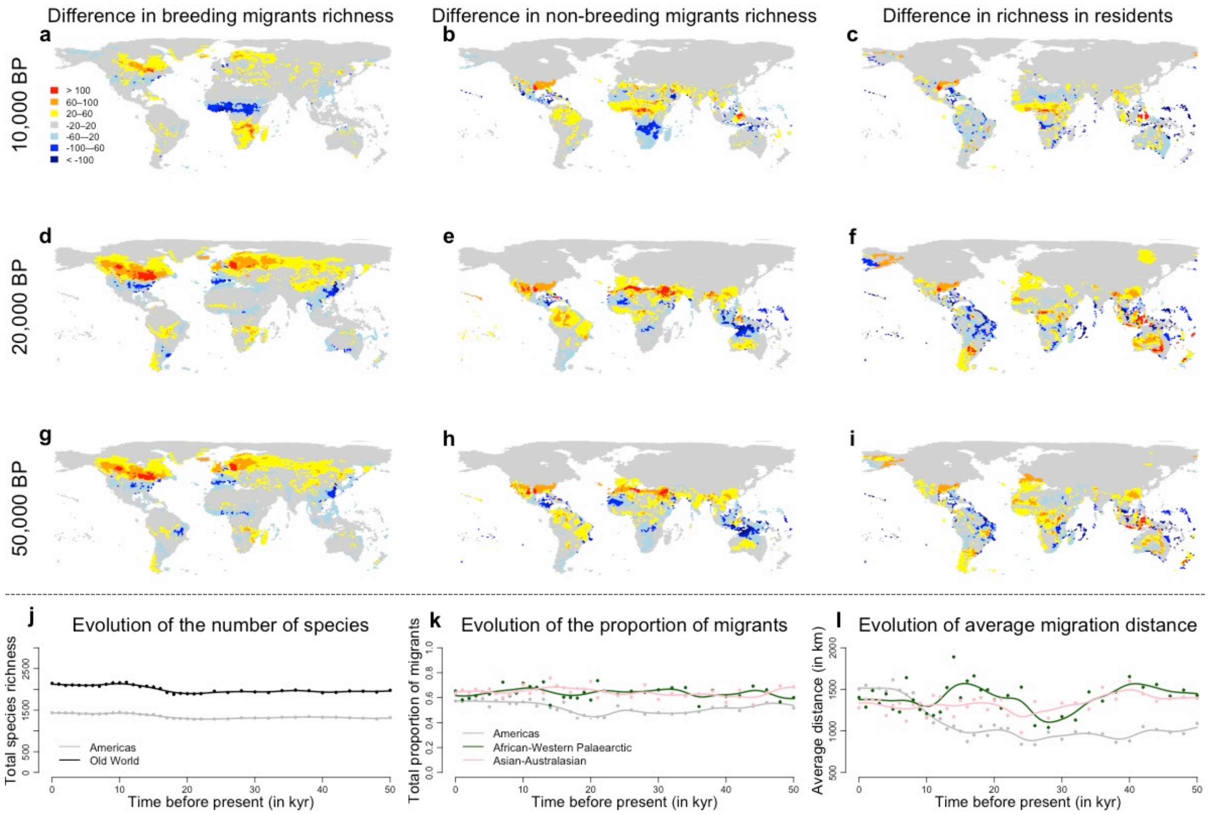


**Supplementary Figure 8:** Reconstruction of the global seasonal distribution of birds for $\beta$ = 0.007. **a**–**i** Predicted contrast between global seasonal patterns of bird diversity (breeding migrants, non-breeding migrants and residents) over the past 50,000 years, compared to current patterns. The patterns were computed as the predicted richness in the past, i.e., predicted number of species per hexagon: 10,000 years before present (**a**–**c**); 20,000 BP (**d**–**f**); and 50,000 BP (**g**–**i**), minus the predicted richness in the present. Red areas had more species than today, blue areas fewer. **j** Predicted evolution of the total number of simulated migratory bird species, which remains largely stable over the past 50,000 years, with some slight decrease around 15,000 years before present. **k** Predicted evolution of the proportion of simulated migratory bird species. **l** Predicted evolution of the average distance between breeding and non-breeding grounds for migrant species, computed as the great circle distance between the centroids of the seasonal ranges. These simulated time series (**j**–**l**) are shown for the Americas (in grey) and the Old World (in black), the former being separated into the African–Western Palaearctic (centroids of non-breeding ranges of simulated species with a longitude < 60°E) and Asian-Australasian (centroids of non-breeding ranges of simulated species with a longitude >= 60°E) regions in panels **k**,**l**. BP: Before Present; 1kyr = 1,000 years.

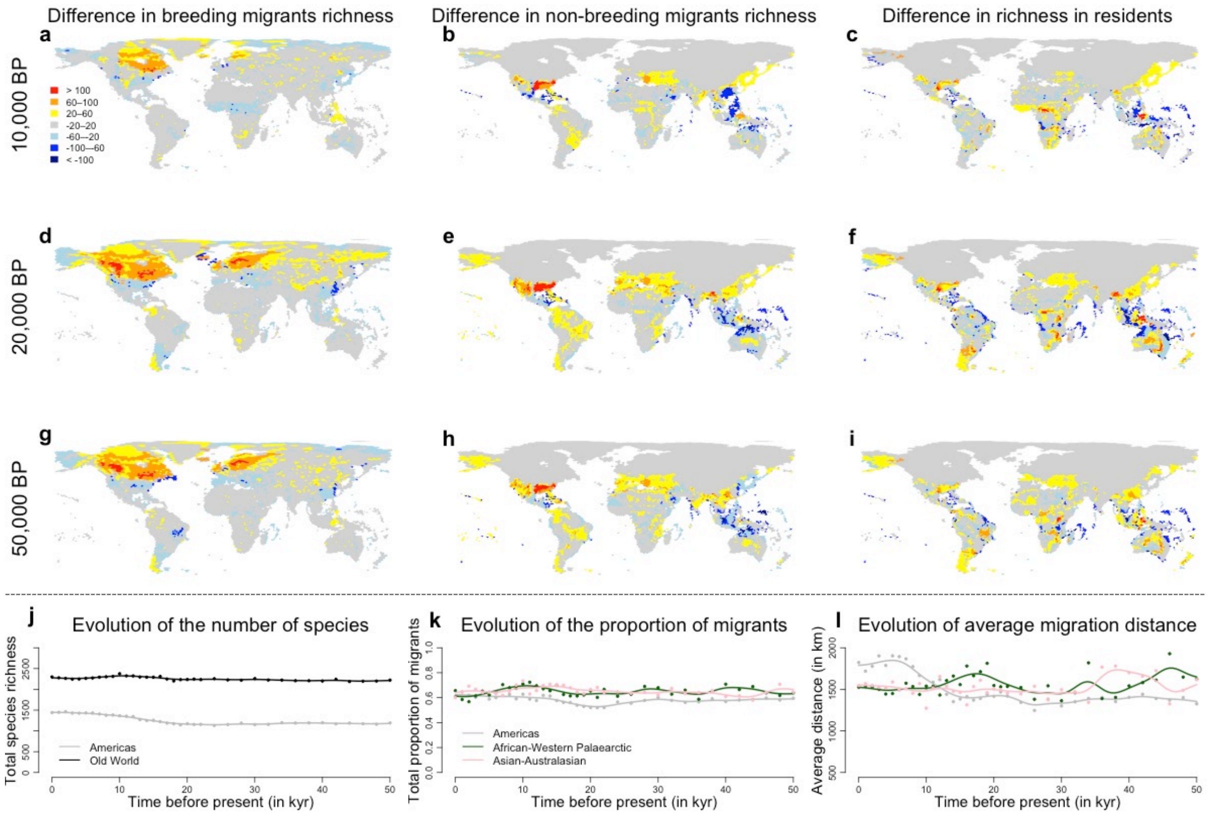


**Supplementary Figure 9:** Reconstruction of the global seasonal distribution of birds for $\beta = 0.02$. **a–i** Predicted contrast between global seasonal patterns of bird diversity (breeding migrants, non-breeding migrants and residents) over the past 50,000 years, compared to current patterns. The patterns were computed as the predicted richness in the past, i.e., predicted number of species per hexagon: 10,000 years before present (**a–c**); 20,000 BP (**d–f**); and 50,000 BP (**g–i**), minus the predicted richness in the present. Red areas had more species than today, blue areas fewer. **j** Predicted evolution of the total number of simulated migratory bird species, which remains largely stable over the past 50,000 years, with some slight decrease around 15,000 years before present. **k** Predicted evolution of the proportion of simulated migratory bird species. **l** Predicted evolution of the average distance between breeding and non-breeding grounds for migrant species, computed as the great circle distance between the centroids of the seasonal ranges. These simulated time series (**j–l**) are shown for the Americas (in grey) and the Old World (in black), the former being separated into the African–Western Palaearctic (centroids of non-breeding ranges of simulated species with a longitude < 60°E) and Asian-Australasian (centroids of non-breeding ranges of simulated species with a longitude >= 60°E) regions in panels **k**,**l**. BP: Before Present; 1kyr = 1,000 years.

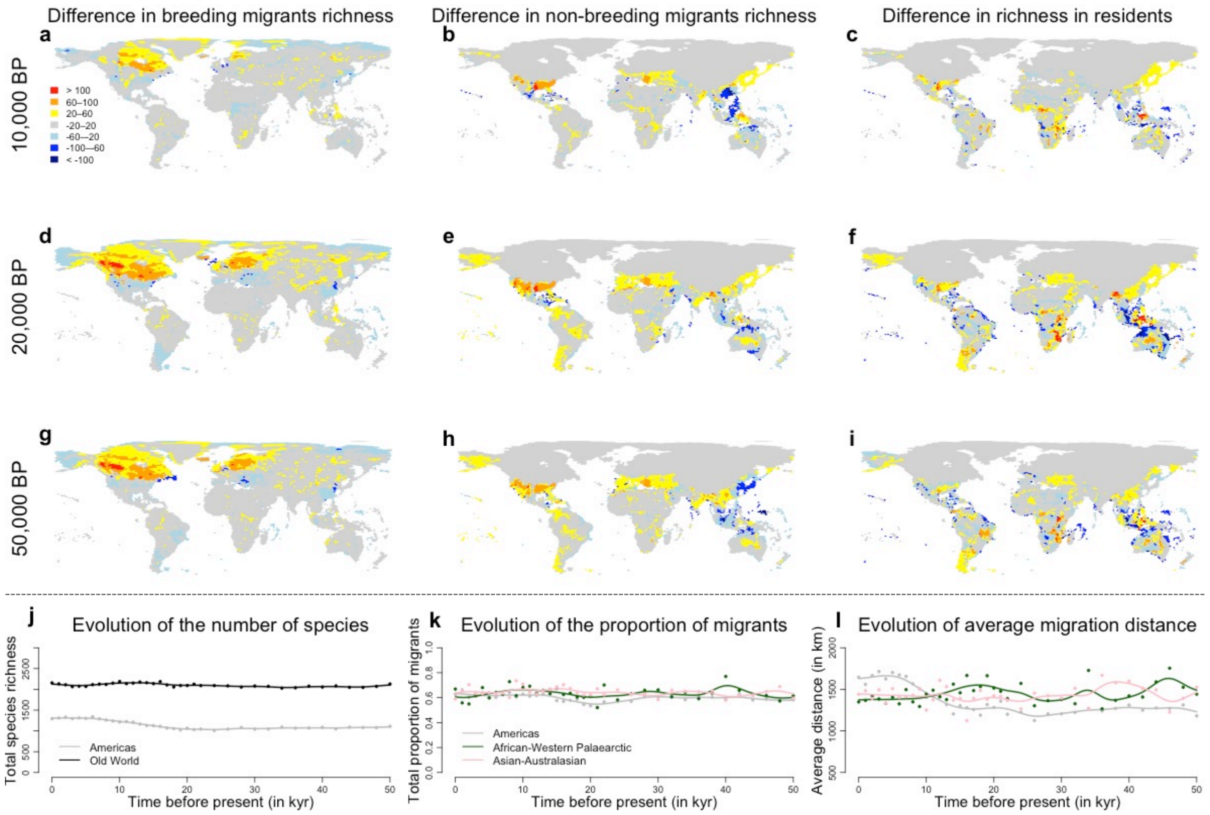


**Supplementary Figure 10:** Reconstruction of the global seasonal distribution of birds for $\beta$ = 0.035. **a–i** Predicted contrast between global seasonal patterns of bird diversity (breeding migrants, non-breeding migrants and residents) over the past 50,000 years, compared to current patterns. The patterns were computed as the predicted richness in the past, i.e., predicted number of species per hexagon: 10,000 years before present (**a–c**); 20,000 BP (**d–f**); and 50,000 BP (**g–i**), minus the predicted richness in the present. Red areas had more species than today, blue areas fewer. **j** Predicted evolution of the total number of simulated migratory bird species, which remains largely stable over the past 50,000 years, with some slight decrease around 15,000 years before present. **k** Predicted evolution of the proportion of simulated migratory bird species. **l** Predicted evolution of the average distance between breeding and non-breeding grounds for migrant species, computed as the great circle distance between the centroids of the seasonal ranges. These simulated time series (**j–l**) are shown for the Americas (in grey) and the Old World (in black), the former being separated into the African–Western Palaearctic (centroids of non-breeding ranges of simulated species with a longitude < 60°E) and Asian-Australasian (centroids of non-breeding ranges of simulated species with a longitude >= 60°E) regions in panels **k**,**l**. BP: Before Present; 1kyr = 1,000 years.

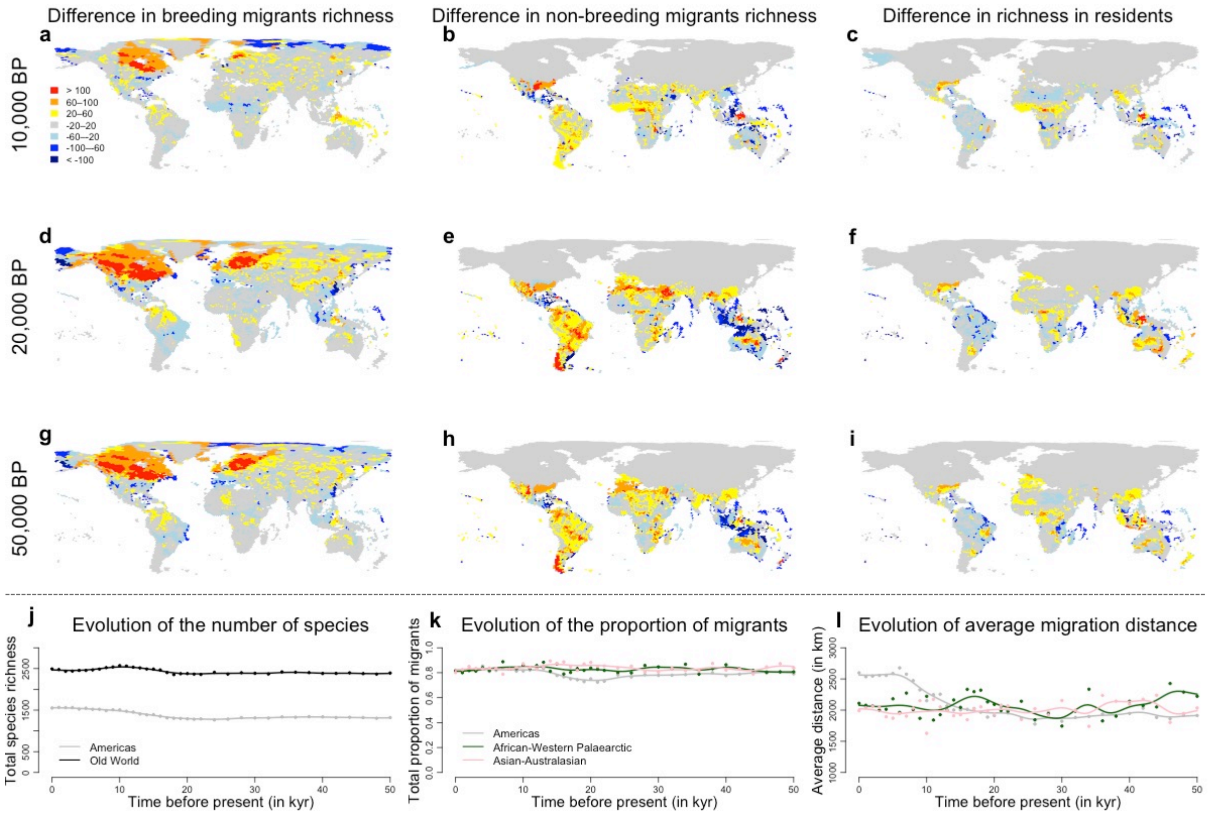


**Supplementary Figure 11:** Reconstruction of the global seasonal distribution of birds for $\alpha$ = 0.00001. **a**–**i** Predicted contrast between global seasonal patterns of bird diversity (breeding migrants, non-breeding migrants and residents) over the past 50,000 years, compared to current patterns. The patterns were computed as the predicted richness in the past, i.e., predicted number of species per hexagon: 10,000 years before present (**a**–**c**); 20,000 BP (**d**–**f**); and 50,000 BP (**g**–**i**), minus the predicted richness in the present. Red areas had more species than today, blue areas fewer. **j** Predicted evolution of the total number of simulated migratory bird species, which remains largely stable over the past 50,000 years, with some slight decrease around 15,000 years before present. **k** Predicted evolution of the proportion of simulated migratory bird species. **l** Predicted evolution of the average distance between breeding and non-breeding grounds for migrant species, computed as the great circle distance between the centroids of the seasonal ranges. These simulated time series (**j**–**l**) are shown for the Americas (in grey) and the Old World (in black), the former being separated into the African–Western Palaearctic (centroids of non-breeding ranges of simulated species with a longitude < 60°E) and Asian-Australasian (centroids of non-breeding ranges of simulated species with a longitude >= 60°E) regions in panels **k**,**l**. BP: Before Present; 1kyr = 1,000 years.

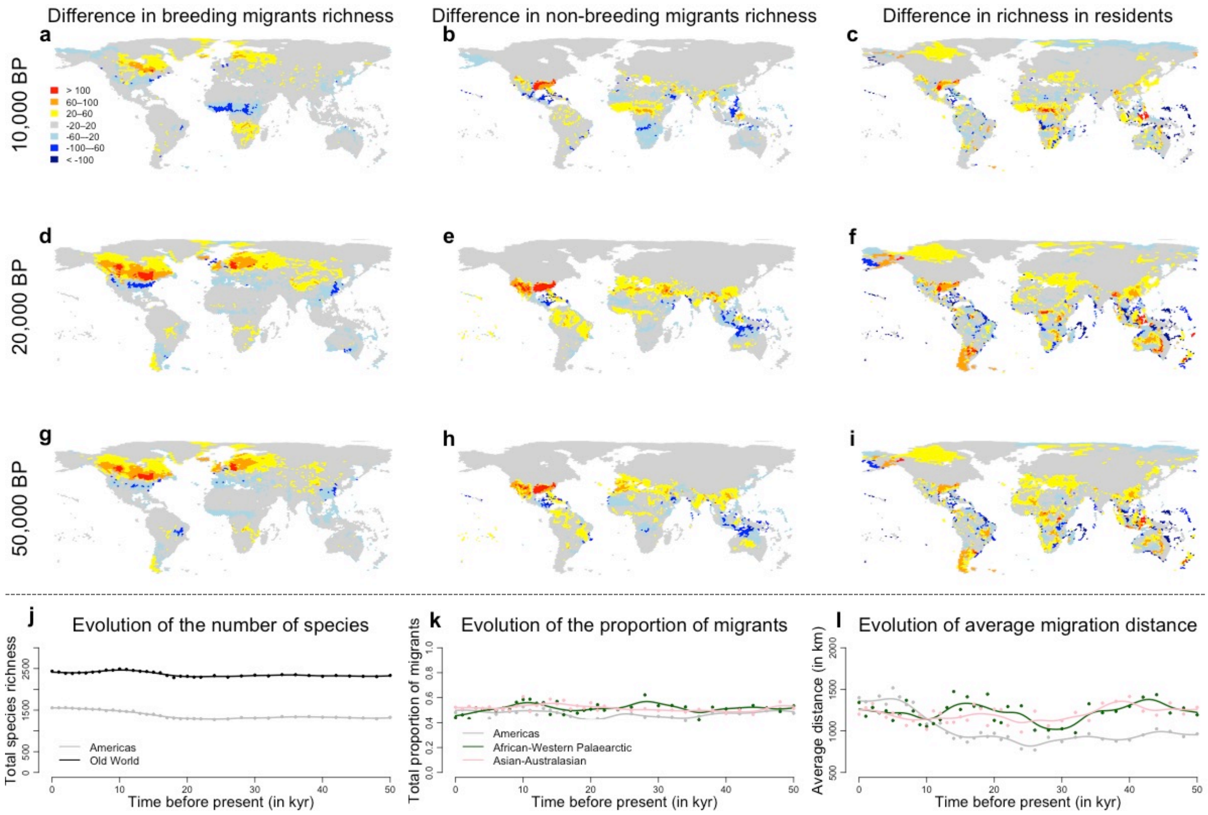


**Supplementary Figure 12:** Reconstruction of the global seasonal distribution of birds for $\alpha = 0.0001$. **a**–**i** Predicted contrast between global seasonal patterns of bird diversity (breeding migrants, non-breeding migrants and residents) over the past 50,000 years, compared to current patterns. The patterns were computed as the predicted richness in the past, i.e., predicted number of species per hexagon: 10,000 years before present (**a**–**c**); 20,000 BP (**d**–**f**); and 50,000 BP (**g**–**i**), minus the predicted richness in the present. Red areas had more species than today, blue areas fewer. **j** Predicted evolution of the total number of simulated migratory bird species, which remains largely stable over the past 50,000 years, with some slight decrease around 15,000 years before present. **k** Predicted evolution of the proportion of simulated migratory bird species. **l** Predicted evolution of the average distance between breeding and non-breeding grounds for migrant species, computed as the great circle distance between the centroids of the seasonal ranges. These simulated time series (**j**–**l**) are shown for the Americas (in grey) and the Old World (in black), the former being separated into the African–Western Palaearctic (centroids of non-breeding ranges of simulated species with a longitude < 60°E) and Asian-Australasian (centroids of non-breeding ranges of simulated species with a longitude >= 60°E) regions in panels **k**,**l**. BP: Before Present; 1kyr = 1,000 years.

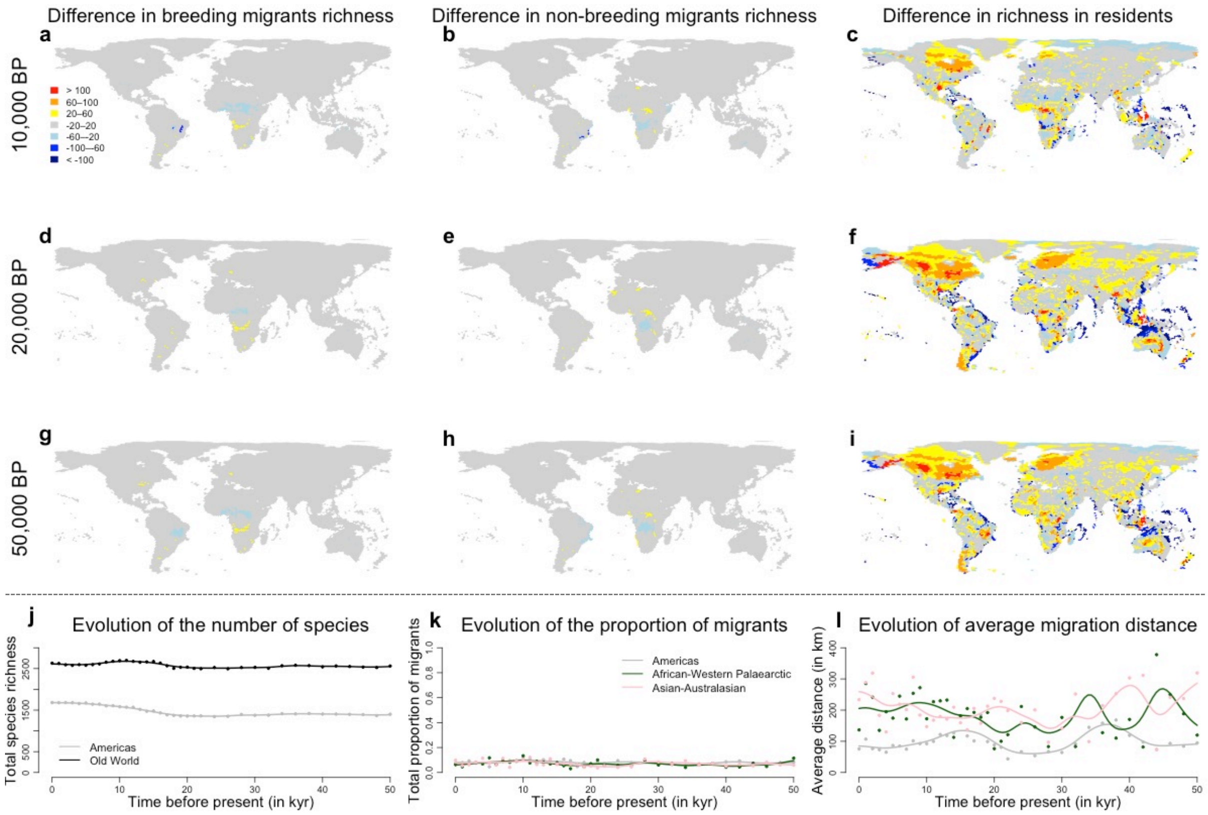


**Supplementary Figure 13:** Reconstruction of the global seasonal distribution of birds for $\alpha = 0.0005$. **a–i** Predicted contrast between global seasonal patterns of bird diversity (breeding migrants, non-breeding migrants and residents) over the past 50,000 years, compared to current patterns. The patterns were computed as the predicted richness in the past, i.e., predicted number of species per hexagon: 10,000 years before present (**a–c**); 20,000 BP (**d–f**); and 50,000 BP (**g–i**), minus the predicted richness in the present. Red areas had more species than today, blue areas fewer. **j** Predicted evolution of the total number of simulated migratory bird species, which remains largely stable over the past 50,000 years, with some slight decrease around 15,000 years before present. **k** Predicted evolution of the proportion of simulated migratory bird species. **l** Predicted evolution of the average distance between breeding and non-breeding grounds for migrant species, computed as the great circle distance between the centroids of the seasonal ranges. These simulated time series (**j–l**) are shown for the Americas (in grey) and the Old World (in black), the former being separated into the African–Western Palaearctic (centroids of non-breeding ranges of simulated species with a longitude < 60°E) and Asian-Australasian (centroids of non-breeding ranges of simulated species with a longitude >= 60°E) regions in panels **k**,**l**. BP: Before Present; 1kyr = 1,000 years.

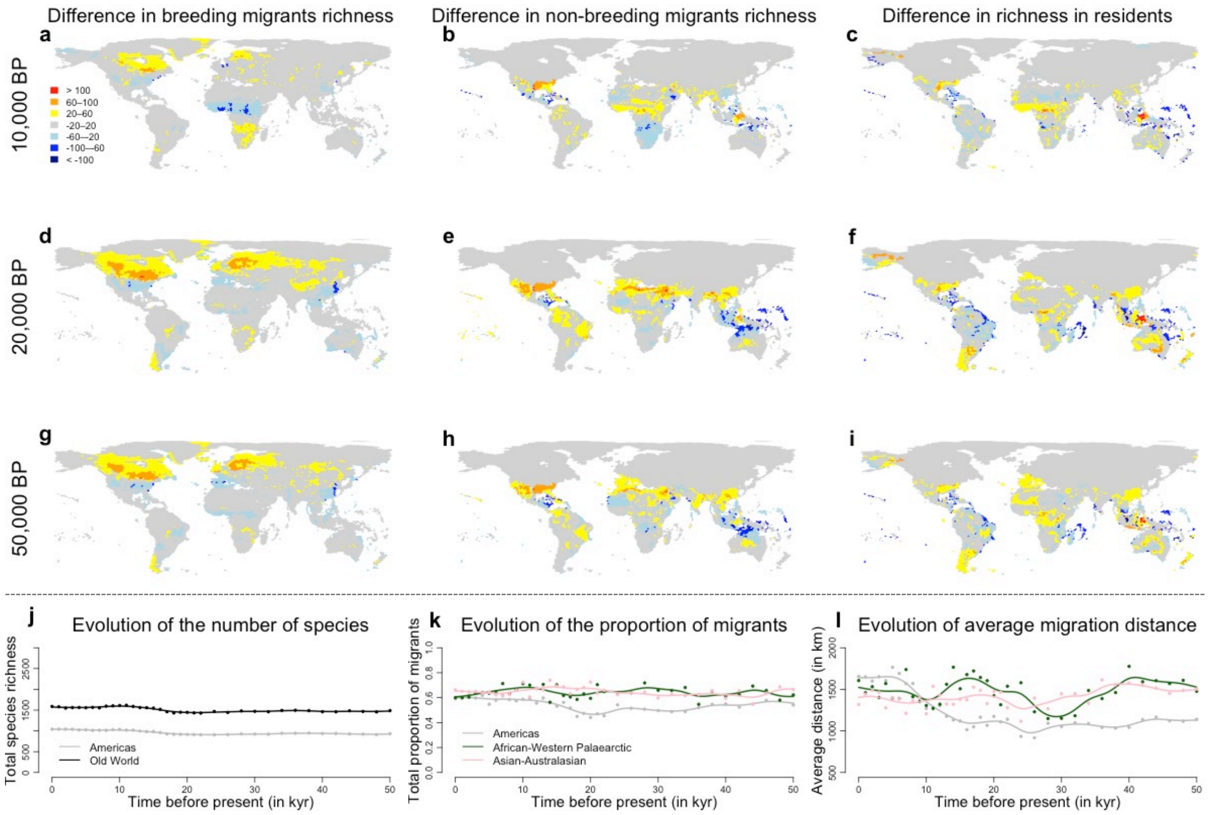


**Supplementary Figure 14:** Reconstruction of the global seasonal distribution of birds for $\mu$ = 45. **a**–**i** Predicted contrast between global seasonal patterns of bird diversity (breeding migrants, non-breeding migrants and residents) over the past 50,000 years, compared to current patterns. The patterns were computed as the predicted richness in the past, i.e., predicted number of species per hexagon: 10,000 years before present (**a**–**c**); 20,000 BP (**d**–**f**); and 50,000 BP (**g**–**i**), minus the predicted richness in the present. Red areas had more species than today, blue areas fewer. **j** Predicted evolution of the total number of simulated migratory bird species, which remains largely stable over the past 50,000 years, with some slight decrease around 15,000 years before present. **k** Predicted evolution of the proportion of simulated migratory bird species. **l** Predicted evolution of the average distance between breeding and non-breeding grounds for migrant species, computed as the great circle distance between the centroids of the seasonal ranges. These simulated time series (**j**–**l**) are shown for the Americas (in grey) and the Old World (in black), the former being separated into the African–Western Palaearctic (centroids of non-breeding ranges of simulated species with a longitude < 60°E) and Asian-Australasian (centroids of non-breeding ranges of simulated species with a longitude >= 60°E) regions in panels **k**,**l**. BP: Before Present; 1kyr = 1,000 years.

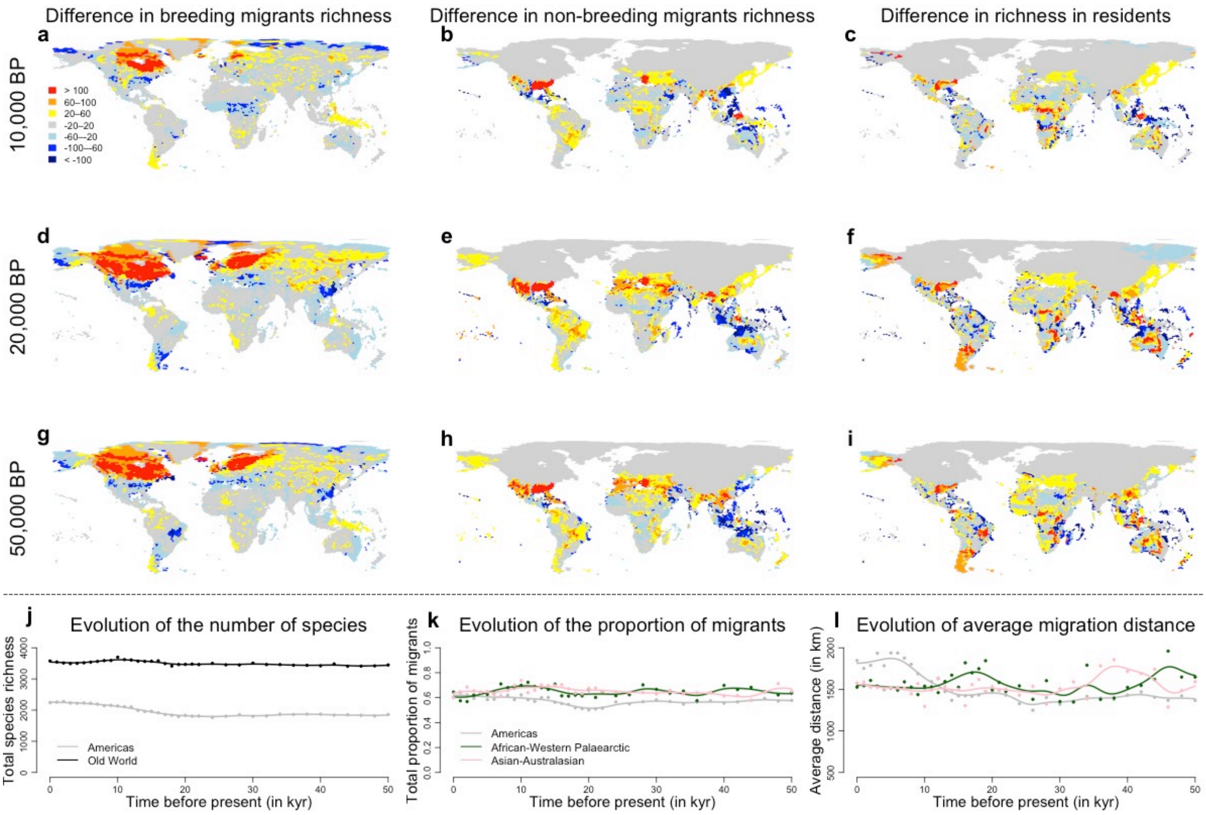


**Supplementary Figure 15:** Reconstruction of the global seasonal distribution of birds for $\mu$ = 100. **a**–**i** Predicted contrast between global seasonal patterns of bird diversity (breeding migrants, non-breeding migrants and residents) over the past 50,000 years, compared to current patterns. The patterns were computed as the predicted richness in the past, i.e., predicted number of species per hexagon: 10,000 years before present (**a**–**c**); 20,000 BP (**d**–**f**); and 50,000 BP (**g**–**i**), minus the predicted richness in the present. Red areas had more species than today, blue areas fewer. **j** Predicted evolution of the total number of simulated migratory bird species, which remains largely stable over the past 50,000 years, with some slight decrease around 15,000 years before present. **k** Predicted evolution of the proportion of simulated migratory bird species. **l** Predicted evolution of the average distance between breeding and non-breeding grounds for migrant species, computed as the great circle distance between the centroids of the seasonal ranges. These simulated time series (**j**–**l**) are shown for the Americas (in grey) and the Old World (in black), the former being separated into the African–Western Palaearctic (centroids of non-breeding ranges of simulated species with a longitude < 60°E) and Asian-Australasian (centroids of non-breeding ranges of simulated species with a longitude >= 60°E) regions in panels **k**,**l**. BP: Before Present; 1kyr = 1,000 years.

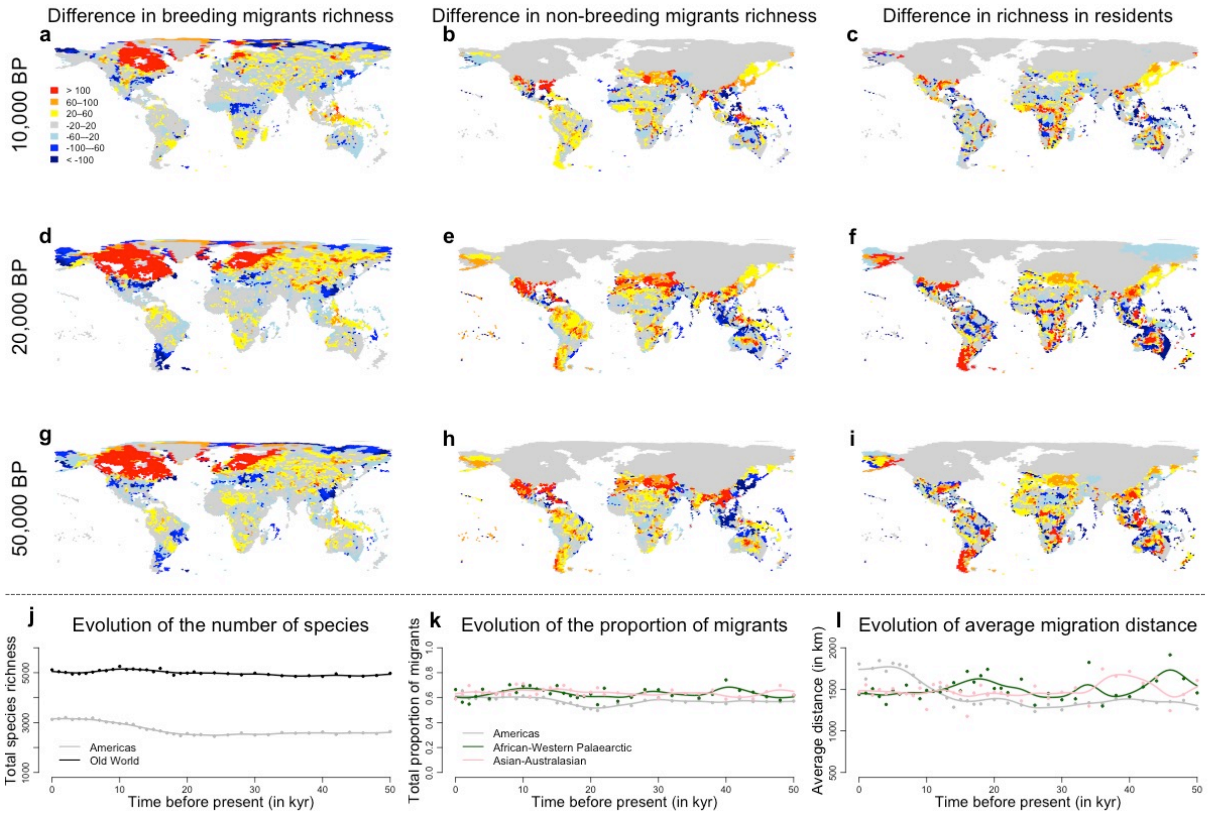


**Supplementary Figure 16:** Reconstruction of the global seasonal distribution of birds for $\mu$ = 150. **a**–**i** Predicted contrast between global seasonal patterns of bird diversity (breeding migrants, non-breeding migrants and residents) over the past 50,000 years, compared to current patterns. The patterns were computed as the predicted richness in the past, i.e., predicted number of species per hexagon: 10,000 years before present (**a**–**c**); 20,000 BP (**d**–**f**); and 50,000 BP (**g**–**i**), minus the predicted richness in the present. Red areas had more species than today, blue areas fewer. **j** Predicted evolution of the total number of simulated migratory bird species, which remains largely stable over the past 50,000 years, with some slight decrease around 15,000 years before present. **k** Predicted evolution of the proportion of simulated migratory bird species. **l** Predicted evolution of the average distance between breeding and non-breeding grounds for migrant species, computed as the great circle distance between the centroids of the seasonal ranges. These simulated time series (**j**–**l**) are shown for the Americas (in grey) and the Old World (in black), the former being separated into the African–Western Palaearctic (centroids of non-breeding ranges of simulated species with a longitude < 60°E) and Asian-Australasian (centroids of non-breeding ranges of simulated species with a longitude >= 60°E) regions in panels **k**,**l**. BP: Before Present; 1kyr = 1,000 years.
