# Supplementary material for: Simulation-based reconstruction of global bird migration over the past 50,000 years
Source: Nat Commun. 2020 Feb 18;11:801. doi: 10.1038/s41467-020-14589-2 (PMC7028998; doi:10.1038/s41467-020-14589-2)
Supplement: Supplementary file 3 — Description of Additional Supplementary Information [file 41467_2020_14589_MOESM3_ESM.docx]

**Description of Additional Supplementary Files**

**File Name**: Supplementary Movie 1
**Description:** Climate reconstruction. This animated figure shows the spatial patterns in climate and productivity from present to 50,000 years ago. Top row: temperature; middle row: precipitation (log[x+1]); bottom row: net primary productivity (NPP; log[x+1]). The column of panels on the right shows the evolution of the median values for temperature, precipitation and NPP across the world and for each season. 1ka = 1,000 years.

**File Name**: Supplementary Movie 2
**Description:** Geographical distribution of simulated range options. For each time slice, the map shows the density of simulated range options across the world. Range options were simulated using the spreading dye algorithm (see Methods for details) and the density in a given hexagon was computed as the number of range options that comprises this hexagon.
