# Supplementary figures and images for: Simulation-based reconstruction of global bird migration over the past 50,000 years
Source: Nat Commun. 2020 Feb 18;11:801. doi: 10.1038/s41467-020-14589-2 (PMC7028998; doi:10.1038/s41467-020-14589-2)

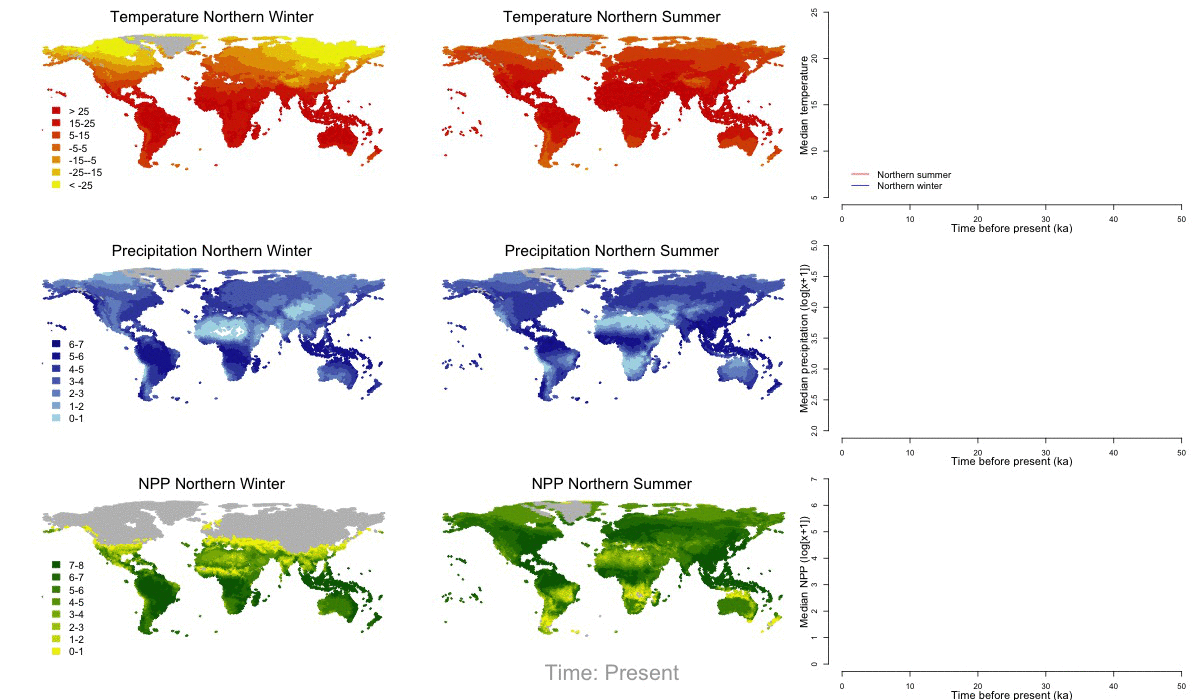

Supplement: Supplementary file 4 — Supplementary Movie 1 [file 41467_2020_14589_MOESM4_ESM.gif]

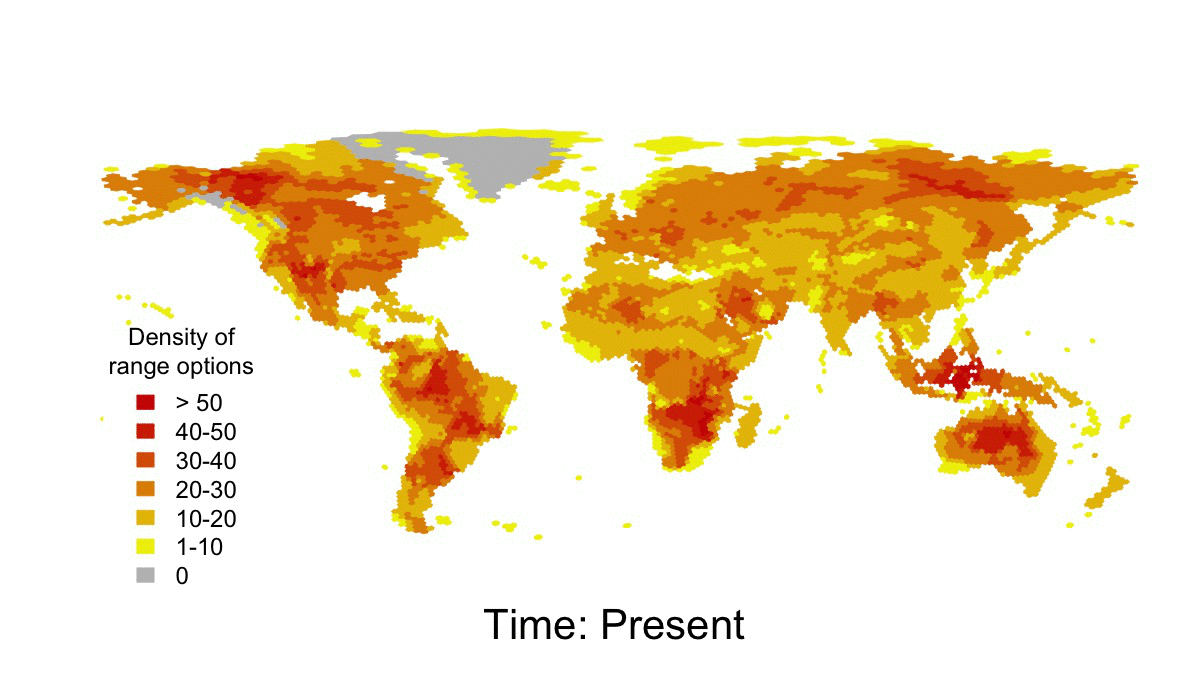

Supplement: Supplementary file 5 — Supplementary Movie 2 [file 41467_2020_14589_MOESM5_ESM.gif]
